# A bibliometric analysis of *Moringa oleifera* during 2000–2024

**DOI:** 10.1515/biol-2025-1314

**Published:** 2026-05-13

**Authors:** Sini Wu, Xiaoyin Li, Guo Zhou, Lijin Pan, Yan Su, Quan Qiu, Junjie Zhang, Qian He

**Affiliations:** College of Forestry and Landscape Architecture, South China Agricultural University, 510642, Guangzhou, China; Guangdong Key Laboratory for Innovative Development and Utilization of Forest Plant Germplasm, College of Forestry and Landscape Architecture, South China Agricultural University, 510642, Guangzhou, China

**Keywords:** *Moringa oleifera*, biblio-metrics analysis, nutritional components, sustainable agriculture

## Abstract

*Moringa oleifera* is a fast-growing, high-yielding tree species rich in bioactive compounds, holding significant potential for production and application across various fields. This review provides a comprehensive overview of the development, hotspots, and future trends in its study over the past 25 years, aiming to provide strategic insights for future research and applications. Using bibliometric tools including VOS-viewer, Cite-Space, and Biblio-Metrix R-package, we analyzed publications from the Web of Science Core Collection (2000–2024). The analysis of the 2,704 publications ultimately collected, with 426 from India and 292 from China, indicates that the research has experienced rapid development over the past five years. The extensive participation of various actors has fostered a complex global collaboration network, where countries/regions leverage their unique resources and expertise to create a complementary research landscape. Research on *M. oleifera* is interdisciplinary. Burst detection, clustering analysis, and thematic evolution analysis revealed that “leaves” and “extraction” will continue to be keywords. Nutritional components, medicinal uses, and water purification have become mature and stable areas. Growth promotion and quality improvement are gradually emerging as core themes. Biofuels and emissions may become new fields of interest. Overall, *M. oleifera* holds great promise for sustainable agriculture, health food, and environmental remediation.

## 1 Introduction

*Moringa oleifera* Lam., belonging to the *Moringaceae* family and the *Moringa* genus, is also known by various names depending on its location and use [1]. Indigenous to the Indian subcontinent, it ranks as the most extensively developed and employed among approximately 14 varieties internationally [2], 3]. *M. oleifera* possesses resilient adaptive features such as being fast-growing, highly adaptable, and drought-resistant, which allows for its widespread cultivation in subtropical and tropical countries [4], 5]. Currently, it grows in the wild or is cultivated in many countries including India, Sri Lanka, and parts of southwestern and northeastern Africa [6].

### 1.1 Bioactive components and applications

Virtually every part of *M. oleifera* exhibits unique utilities [7], covering leaves, roots, seeds, flowers, etc., and is extensively applied across various domains such as food, medicine, feed, and beyond. To clearly and systematically summarize the distinct bioactive properties and multifunctional applications of each plant part, a detailed overview is provided in Table 1.

**Table 1:** Overview of bioactive components and applications of *Moringa oleifera.*

Part of plant	Major bioactive components and characteristics	Core biological functions/activities	Practical applications and value	Revised reference
Leaves	High content of proteins, vitamins, minerals, essential amino acids, flavonoids, and plant hormones	Antioxidant, anti-inflammatory, blood glucose/lipid regulation, liver protection, and improvement of malnutrition	1. Nutrition and food: nutritional supplements, food fortifiers. 2. Medicine and Health: raw materials for pharmaceuticals/functional foods, used for anemia and chronic disease management. 3. Feed and livestock: high-protein feed additives, improving growth performance and product quality. 4. Agriculture: extracting zeatin as foliar fertilizer to increase crop yield	[8], 9], [11], [12], [13], [14, 26], 27]
Seeds	Rich in oils (high in behenic acid) and protein peptides with flocculation activity	Antibacterial, anti-inflammatory, efficient water purification	1. Industry and water treatment: natural biodegradable flocculants for drinking water purification. 2. Food and cosmetics: extracting edible oil, oil for cosmetics. 3. Energy: potential biofuel feedstock	[5], 29]
Root/bark	Contains alkaloids, phenolic compounds, etc	Traditionally used for anti-inflammatory, analgesic, and ulcer treatment	Medicine and health: mainly used in traditional medicine formulations, with limited modern applications, requiring cautious development	[3]
Flowers	Rich in free amino acids, flavonoid compounds, and nutritionally dense	Antioxidant, hepatoprotective, traditionally used as a diuretic and tonic	1. Nutrition and food: edible as vegetables or herbal tea. 2. Medicine and health: raw materials for health products or herbal preparations	[7]
Pods	Extremely high vitamin C content, rich in fiber and other minerals	Provides high doses of vitamin C, antioxidant, promotes digestion	Nutrition and food: directly consumed as a high vitamin C vegetable (common in dishes in India, Africa, etc.)	[6]
Whole plant	Fast-growing, high biomass, strong adaptability	Carbon sequestration, soil improvement, soil and water conservation	Environment and sustainable agriculture: used for carbon sequestration, as an agroforestry species to improve the ecological environment	[4], 5], 28]

### 1.2 Multifunctional value and integrated applications

**Nutritional value**. As highlighted in Table 1, each part of *M. oleifera* harbors high nutritional value and low levels of anti-nutritional factors. This plant contains high levels of minerals like calcium, iron, potassium, zinc, and copper [8], along with proteins, amino acids, and diverse vitamins, all present in concentrations several times greater than in typical fruits and vegetables [9]. Additionally, *M. oleifera* contains a variety of rare and beneficial compounds, including beta-sitosterol, quercetin, zeatin, caffeoylquinic acid and kaempferol [10], all of which are essential for human health. Among these parts, leaves are the most nutritious and commonly used part of the *M. oleifera* tree. They are excellent sources of concentrated protein, vitamins and minerals [11], 12]. Additionally, immature pods are widely consumed in South Africa as a food with an extremely high vitamin C content.

**Medicinal value**. The medicinal properties of *M. oleifera* are largely due to its unique composition of phytochemicals [13], as listed in Table 1. It is recognized as a natural, reliable, and safe source when used at established concentrations. Several studies have demonstrated that *M. oleifera* plays a significant role in improving malnutrition, treating anemia, and providing anti-cancer, antioxidant, anti-diabetic, anti-inflammatory, anti-bacterial effects. It also protects the nervous system and supports liver and kidney health [14], [15], [16], [17], [18]. The bioactive compounds in *M. oleifera* have been shown to contribute significantly to its pharmacological effects. Owing to its remarkable medicinal properties, *M. oleifera* is often referred to as the “Miracle Tree”, “Panacea”, and “Medicine Treasure Chest.”

**Feeding value**. The abundant bioactive compounds and nutrients also offer great potential as a livestock feed resource. Compared with the traditional feed, the woody forage represented by *M. oleifera* has several advantages, including faster growth, stronger resistance, higher nutrients content, especially N content, and better palatability [19], 20]. These advantages pertain to the plant itself as a feed source. As a novel protein feed resource, multiple parts can be used directly or as an additive, improving the health and growth performance of various livestock [21], a conclusion confirmed in studies involving fish, dairy cows, goats, and hens [14], [22], [23], [24], [25].

*Moringa oleifera* also holds potential applications in other areas. *M. oleifera* seeds are used for water purification and extracting ben oil, which is rich in behenic acid. Moringa leaf extract (MLE), an important natural plant growth promoter, is promoted in sustainable agricultural strategies [26]. Zeatin from the leaves has been developed into a foliar fertilizer that can increase crop yields by 25–30 % [27]. This compound is also used as a raw material in the cosmetics and calico-printing industries. Moreover, the potential of *M. oleifera* for carbon storage is being continually explored [28], which can help mitigate the adverse effects of climate change. Notably, in recent years, Studies indicate that *M. oleifera* seeds are a prospective source of biofuel, capable of replacing traditional fossil fuels [29].

In summary, *M. oleifera* is a highly productive and versatile woody plant with broad application prospects. Although the first international conference on *M. oleifera*, held in Tanzania in 2001 [30], brought increased attention to the plant, it is still considered an overlooked and underutilized species. Therefore, it is necessary to systematically review the research progress on *M. oleifera* and propose future strategies to fully utilize this multipurpose plant.

Bibliometric methods are statistically reliable due to the large volume of data [31] and their advantages in social network and co-word analyses. Currently, the utilization of bibliometrics in research on *M. oleifera* is relatively scarce. Therefore, we collected articles on *M. oleifera* from the Web of Science, a core database recognized for its authority in scientific statistics and evaluation, and conducted a comprehensive analysis using bibliometric methods. Through cluster analysis and theme mining, the current research status and future development trends can be determined more intuitively, accurately, and comprehensively. This provides valuable directions and references for future research on *M. oleifera*.

## 2 Research methodology

### 2.1 Data collection

We conducted a literature search on 1st January 2025, using the Web of Science Core Collection (WoSCC) database as the data source. The search covered the period from 1st January 2000 to 31st December 2024. The search string used was TI = (“*M. oleifera*”). We restricted the document type to “article” and “review”, and selected “English” as the language. Considering these are among the most commonly used search types in bibliometrics, they typically encompass the majority of research articles in relevant fields and provide more in-depth analysis. Each search result was manually screened to exclude documents that did not meet the search criteria or had low relevance to the topic. Duplicates were removed by comparing titles and abstracts to ensure that only unique documents were included in the analysis. The criteria for manual screening included assessing the relevance of the document’s title and abstract to the research question, the document’s publication date, and the document’s type to confirm that it met our predefined standards.

A total of 2,704 valid papers were ultimately retrieved. All information from these documents was exported from the WoSCC database in plain text format, including authors, affiliated institutions, countries, titles, keywords, publication years, journals, references, etc. These data have been organized for subsequent bibliometric analysis and visualization plotting.

### 2.2 Research methodology

Biblio-metrics, first introduced by British scientist Pritchard in 1969 as a sub-discipline of library and information science [32], uses data mining, information processing, and visual graphing to perform qualitative and quantitative analyses of published literature. This is essential for evaluating the current state and predicting trends in scientific fields [33], 34]. Widely applied across disciplines like artificial intelligence, management, medicine, and agronomy, bibliometrics has shown significant results [35], [36], [37], [38]. It quantitatively examines author collaboration networks, research impact, institutional contributions, and national contributions to research, identifying publication and research trends [39], 40]. Various visualization tools, including VOS-viewer, Sci-MAT, Bib-Excel, Cite-Space, Hist-Cite, Pajek, Gephi, and the Biblio-metrix R-package [41], 42].

This study primarily employs three visualization software tools: Citie-Space (Version 6.4.R1), VOS-viewer (Version 1.6.20), and Biblio-metrix R-package (Version 4.1.3). Each tool possesses its own unique features and advantages, and this research provides detailed information on the main thresholds used for different analyses with these tools, as well as the number of key nodes selected in sequence.

Citie-Space (https://citespace.podia.com/) (accessed on 5th January 2025) enables easy quantitative and qualitative research on scientific subject areas, visualizing the structure and patterns of domain knowledge [43]. It highlights key pathways and knowledge turning points in the evolution of subject areas.

VOS-viewer (https://www.vosviewer.com/) (accessed on 3rd January 2025) is a software tool developed by the Centre for Science and Technology Studies (CWTS) at Leiden University in the Netherlands for constructing and visualizing bibliometric networks [44]. It focuses on aggregate-level analysis [45] and evaluates research hotspots in the literature through clustering observation, observation density, and coverage observation.

The Biblio-metrix R-package (https://www.bibliometrix.org) (accessed on 8th January 2025) is an R-tool for comprehensive science mapping analysis [46]. Since R is a highly expandable language and development environment, Bibliometrix, which is written in R, has clear advantages in areas such as network analysis and knowledge graphs. Its flexibility makes it particularly useful in the evolving field of bibliometrics [47], 48].

Based on advanced literature search and screening, this study primarily employs bibliometric analysis methods to conduct visual analysis and knowledge mapping of research on *M. oleifera* in the database. Simultaneously, we perform statistical analysis and comprehensive organization of literature information. We utilized VOS-viewer to visualize collaboration and co-authorship networks in related research, and Cite-Space for keyword burst detection and citation burst analysis in references. Furthermore, we used Biblio-metrix R-package to determine the basic characteristics of publications and created strategy and thematic evolution maps for the research on *M. oleifera*. Ultimately, through visual analysis and systematic combing, we accurately ascertain the research hotspots, research status, and frontier trends in different periods. This helps relevant researchers to understand the development of the field and based on this, propose research aspects that should be strengthened in the future.

## 3 Initial data statistics

**Figure 1:**
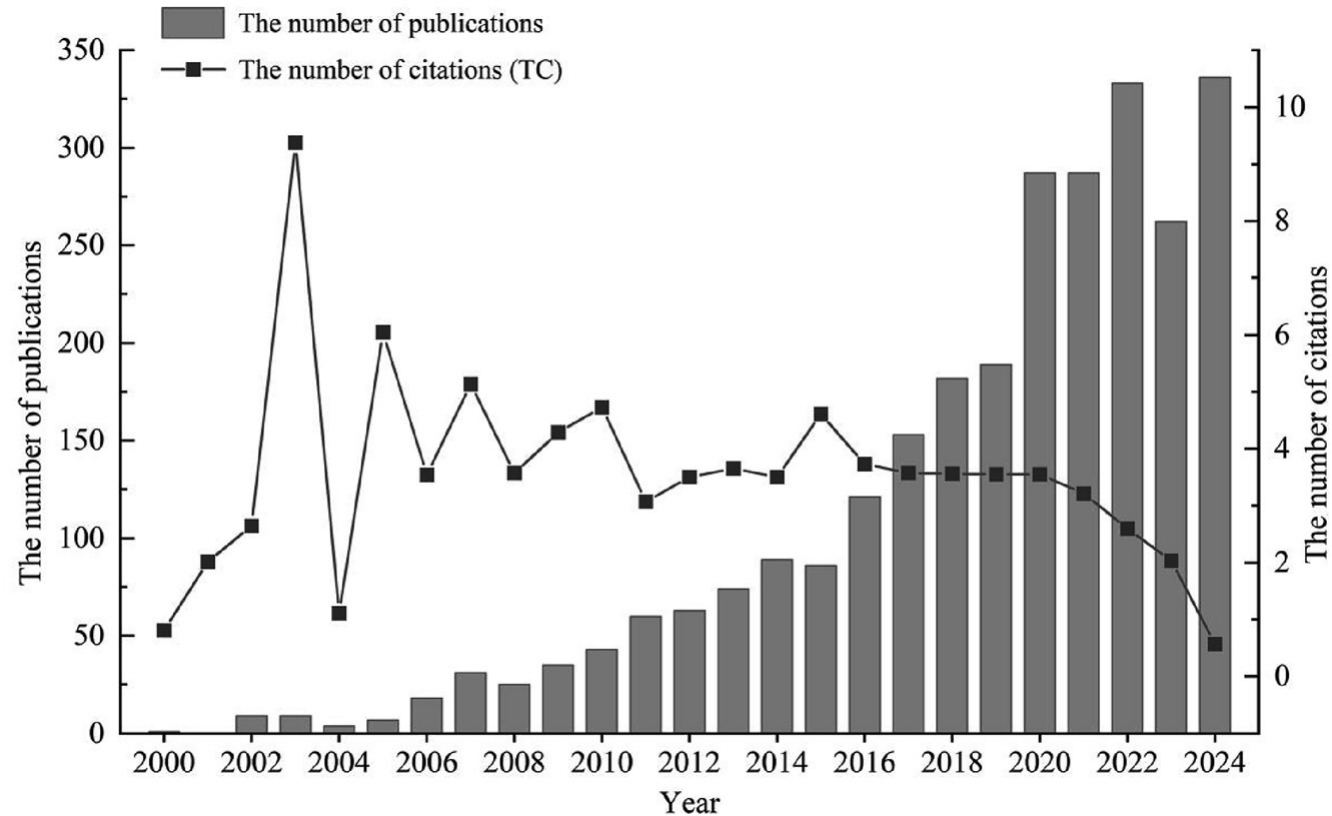
Annual trend of publications and citations related to *Moringa oleifera* in 2000–2024. Grey bars represent the number of publications each year, and the black line with square markers indicates the number of citations. This figure illustrates the increasing research interest in *M. oleifera* over the years.

Figure 1 illustrates the annual trend and total citations for publications related to *M. oleifera* from 2000 to 2024, revealing a general upward trend in the number of annual publications. Based on the annual publications and growth rate, we have categorized the development into four distinct stages: the Germination Stage (2000–2005), the Fluctuating Growth Stage (2006–2014), the Stable Growth Stage (2015–2019), and the Rapid Growth Stage (2020–2024). During the first stage, the number of annual publications did not exceed 10, although the citation rate in 2003 was relatively high. Between 2006 and 2014, significant fluctuations in both the growth rate and citation rate reflected the instability of research output. In the third period, the number of publications related to *M. oleifera* and the average citation counts exhibited a stable growth trend. Despite the fluctuations observed after 2020, which may related to the impact of the COVID-19 pandemic on research activities, the number of publications remained at a relatively high level, with peak annual publication count reaching 336. Although the citation rate has gradually declined, research on *M. oleifera* is currently in a phase of diversification and expansion into new interdisciplinary applications, yet it continues to hold sustained research value and potential.

## 4 Bibliometric analysis

### 4.1 Authors and institutes analysis

Among researchers in the field of *M. oleifera*, several authors have emerged as leaders due to their significant contributions. Bergamasco R. stands out as the most prolific author in terms of overall contributions, with 57 publications and 1,849 citations. Paiva P.M.G. has the highest number of publications, with a total of 60 articles, closely followed by Coelho L.C.B.B. Both authors are also among the earliest to publish research findings on *M. oleifera* and have maintained a consistent output over time. The starting research years of these 10 active authors are mostly within the period of 2006–2015, which helps explain the steady growth phenomenon observed during that time.

In the realm of *M. oleifera* research, several institutions stand out for their prolific publication records. The Egyptian Knowledge Bank (EKB), one of the world’s largest digital library [49], leads the field with 590 aggregated publications, accounting for 21.81 % of all research outputs. Following the EKB are Yunnan Agricultural University, Universidade Estadual de Maringá, and King Saud University.

### 4.2 Journals analysis

**Figure 2:**
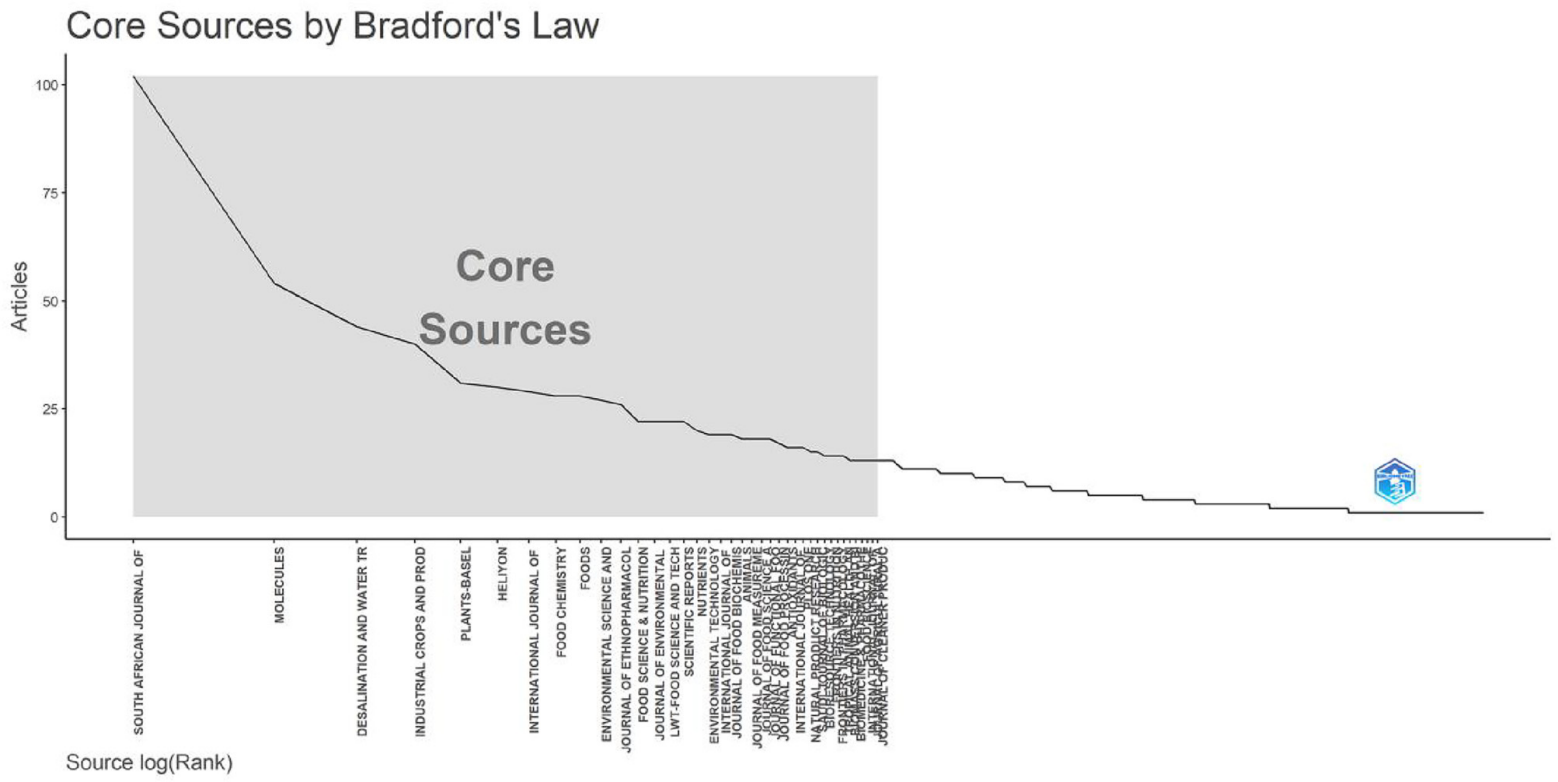
Application of Bradford’s law in *Moringa oleifera* research. This chart illustrates the distribution of articles across various sources following Bradford’s law. The *x*-axis shows source rank, and the *y*-axis shows the number of articles. The grey area indicates core sources, pinpointing the most influential journals in *M. oleifera* research.

Bradford’s Law is a bibliometric principle that describes the distribution of articles across journals in a specific field [50]. It states that if journals are ranked by the number of articles they contain on a particular subject, they can be divided into a core group that contains a significant proportion of the articles, and several successive groups that each contain half the number of articles as the previous group. We applied Bradford’s Law (Figure 2) to identify the most influential journals in the field of *M. oleifera* research, ultimately determining 39 core journals. The five most active journals are as follows: South African Journal of Botany, Molecules (ISSN: 1420–3049), Desalination and Water Treatment (ISSN: 1944–3994), Industrial Crops and Products (ISSN: 0926–6690), and Plants-Basel (ISSN: 2223–7747).

### 4.3 Countries/regions analysis

**Figure 3:**
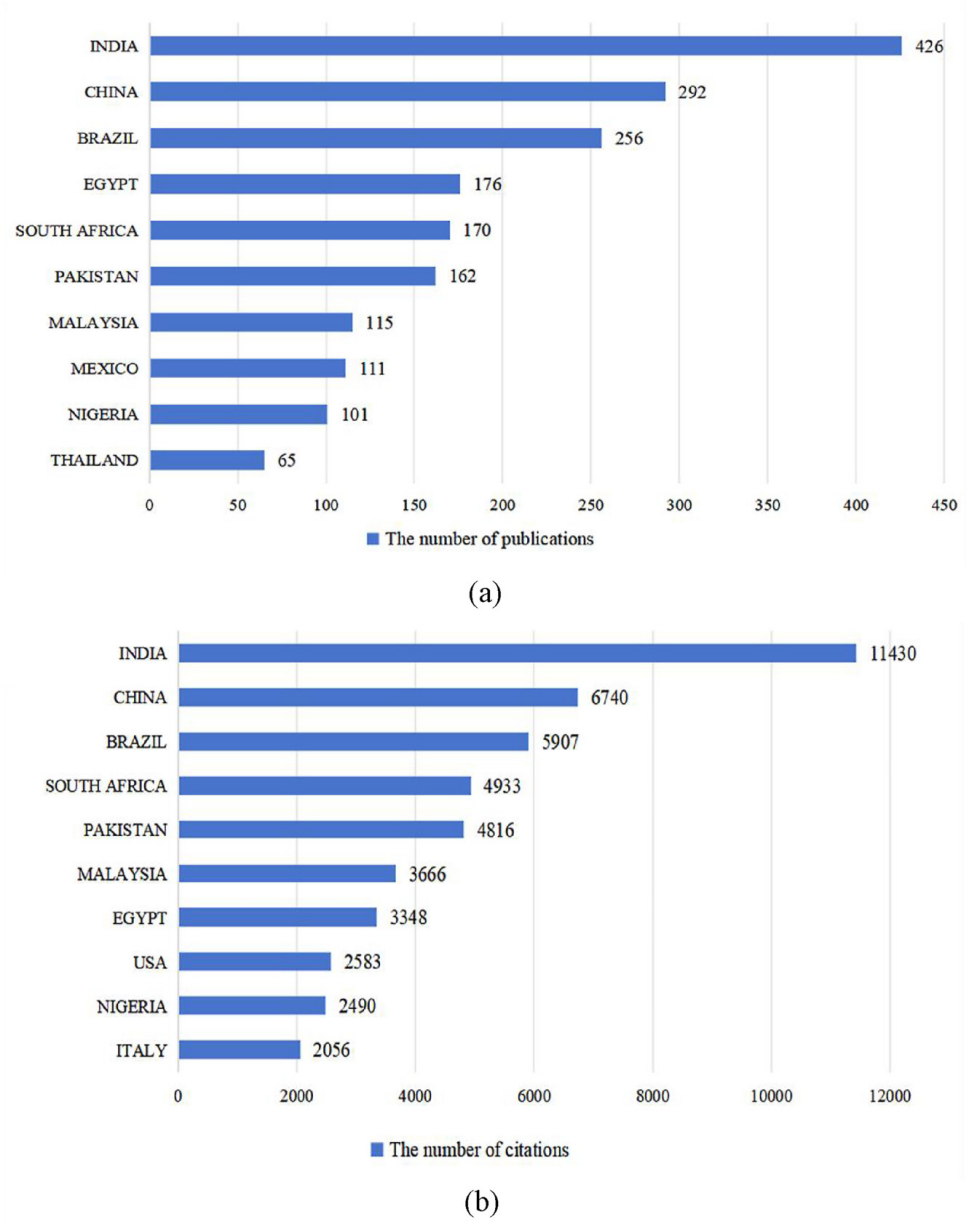
The number of publications (a) and citations (b) in the top 10 countries/regions. Bars represent the count for each country.

Figure 3 indicates that from 2000 to 2024, research on *M. oleifera* was conducted in 95 different countries/regions. The top 10 countries (Figure 3a) and regions (Figure 3b) in terms of publication output accounts for approximately 69.30 % of the global research contributions in this field, with a total of 1,874 publications. India stands out as the largest contributor, with 426 publications and 11,430 citations, closely followed by China (*n* = 292, TC = 6,740) and Brazil (*n* = 256, TC = 5,907). It is worth noting that Mexico and Thailand have entered the top 10 with a high number of publications, while the USA and Italy are characterized by their higher citation counts.

## 5 Network analysis of publications

### 5.1 Institutes collaboration network analysis

**Figure 4:**
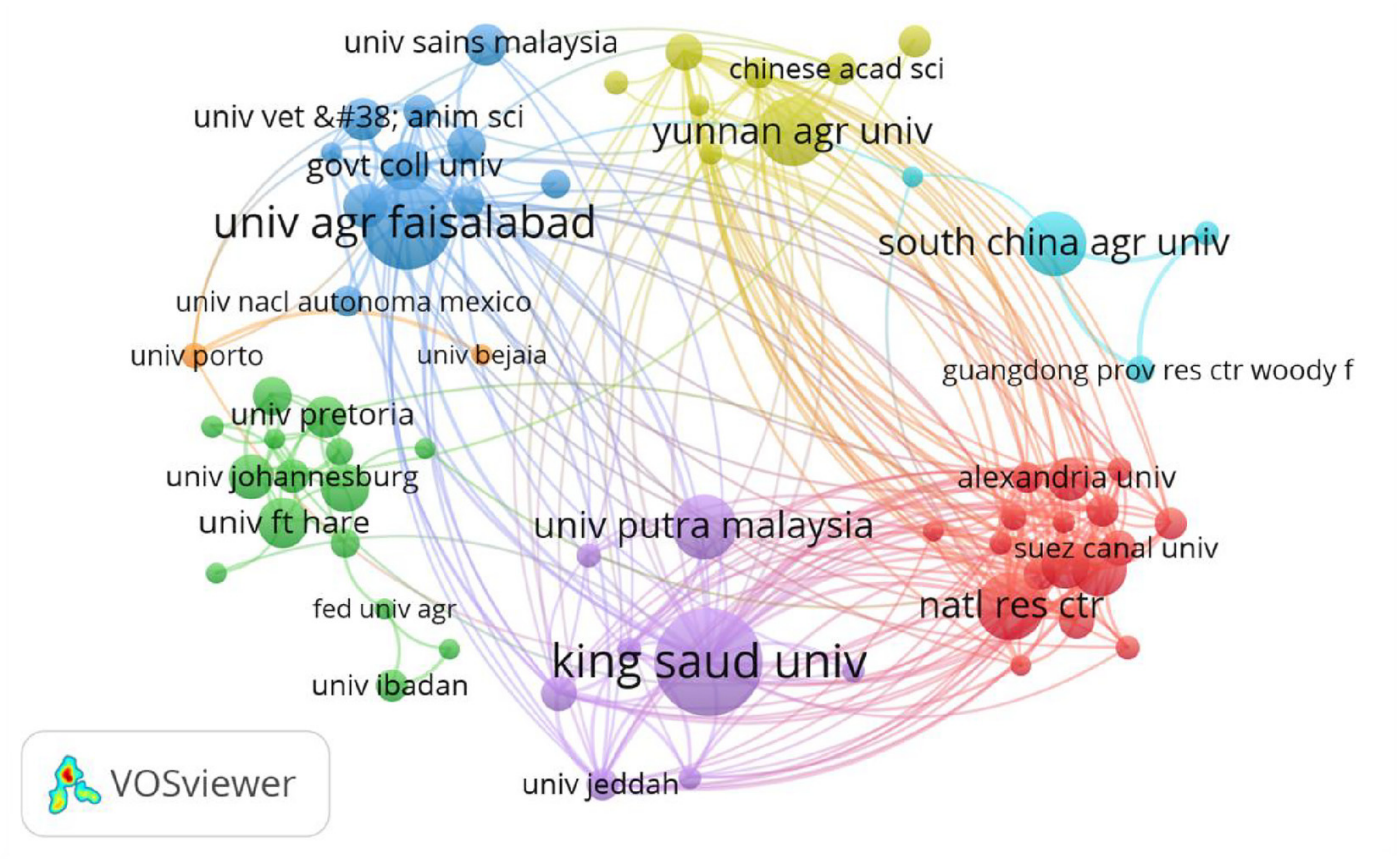
Timeline visualization of institution collaboration network. Node size indicates the number of publications by each institution, and line thickness and distance reflect the extent of collaboration between them. Different colors represent distinct clusters.

The visualization in Figure 4 presents the timeline of the institution cooperation network. The size of the nodes corresponds to the number of publications by each institution, while the thickness and distance of the connecting lines indicate the extent of collaboration between institutions. We established a criterion that the minimum number of documents published by an institution must be at least 10. Ultimately, we screened out 82 institutions from a total of 2,892 research institutes and divided them into five clusters based on citation frequency, using different colors to distinguish each cluster. There are significant differences in the number of collaborations within the institutional collaboration network. King Saud University is an institution with the most collaborative relationships, maintaining close connections with numerous research institutes and have the highest number of citations. The University of Agriculture Faisalabad and the National Research Centre follow closely behind.

### 5.2 Journals collaboration network analysis

**Figure 5:**
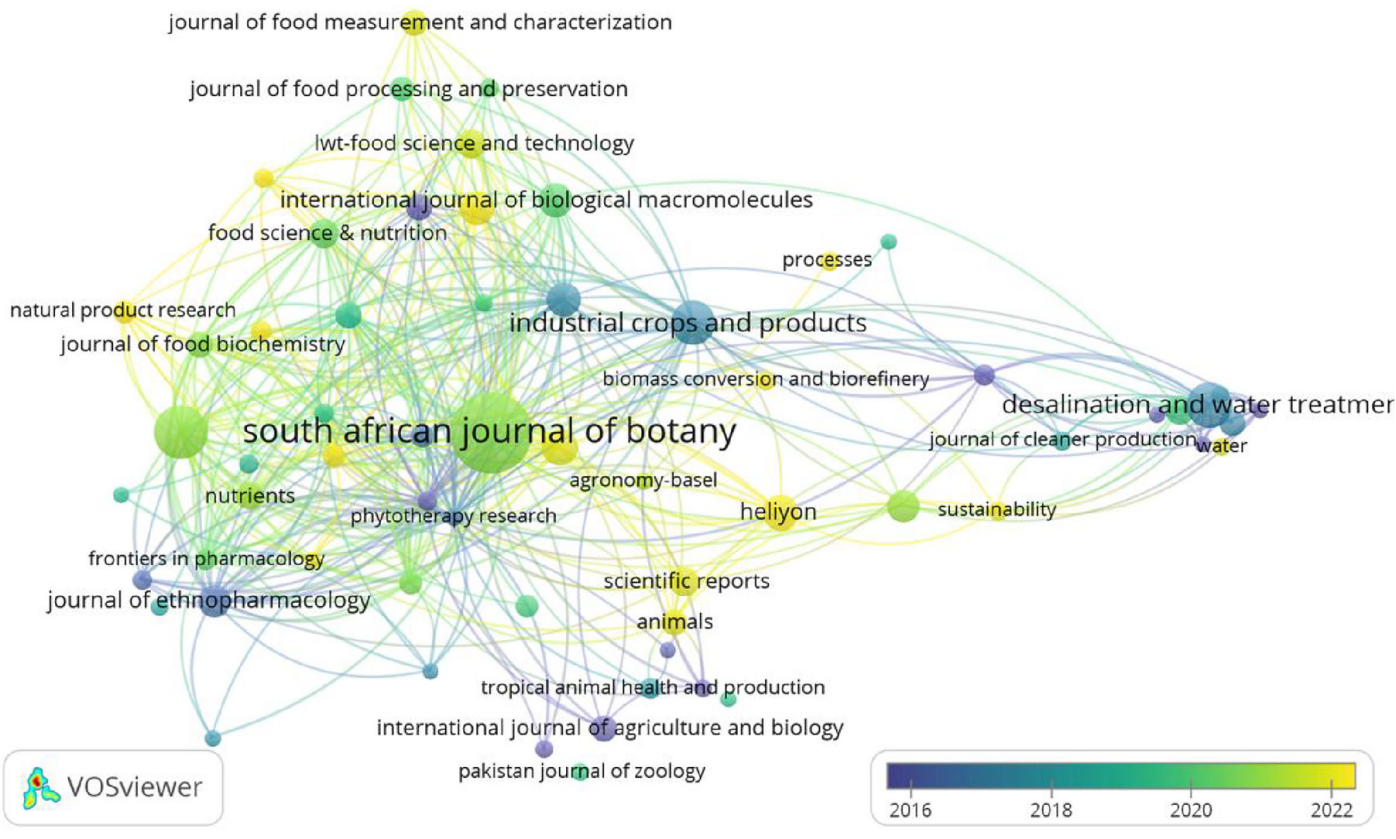
Timeline visualization of journal collaboration network. Node size reflects the number of publications, and color indicates publication year. Lines show collaboration between journals.

Figure 5 illustrates the journal collaboration network in *M. oleifera* research, highlighting significant partnerships through a minimum citation threshold of 10, with ultimately 62 journals meeting this threshold out of 768. The South African Journal of Botany, with a link strength of 901, is a central node, emphasizing its role in disseminating research and fostering academic dialogue on *M. oleifera*. This centrality indicates the journal’s function as a repository for high-quality research and a hub for interdisciplinary exchange. The increase in connection density post-2020, depicted by the color gradient, reflects a growing trend in collaborative research. This analysis identifies key journals driving the field and highlights the importance of aligning research with current trends for impactful publication. It also strategically positions the research of scholars in the field within the academic landscape, emphasizing the benefits of engaging with prominent journals to enhance research visibility and impact. By collaborating with these journals, scholars ensure their contributions are integrated into the scientific discourse on *M. oleifera*. The journal collaboration network analysis provides insights into influential journals, collaborative research trends, and opportunities to enhance the dissemination and impact of findings in the *M. oleifera* field.

### 5.3 Country/regional cooperation analysis

**Figure 6:**
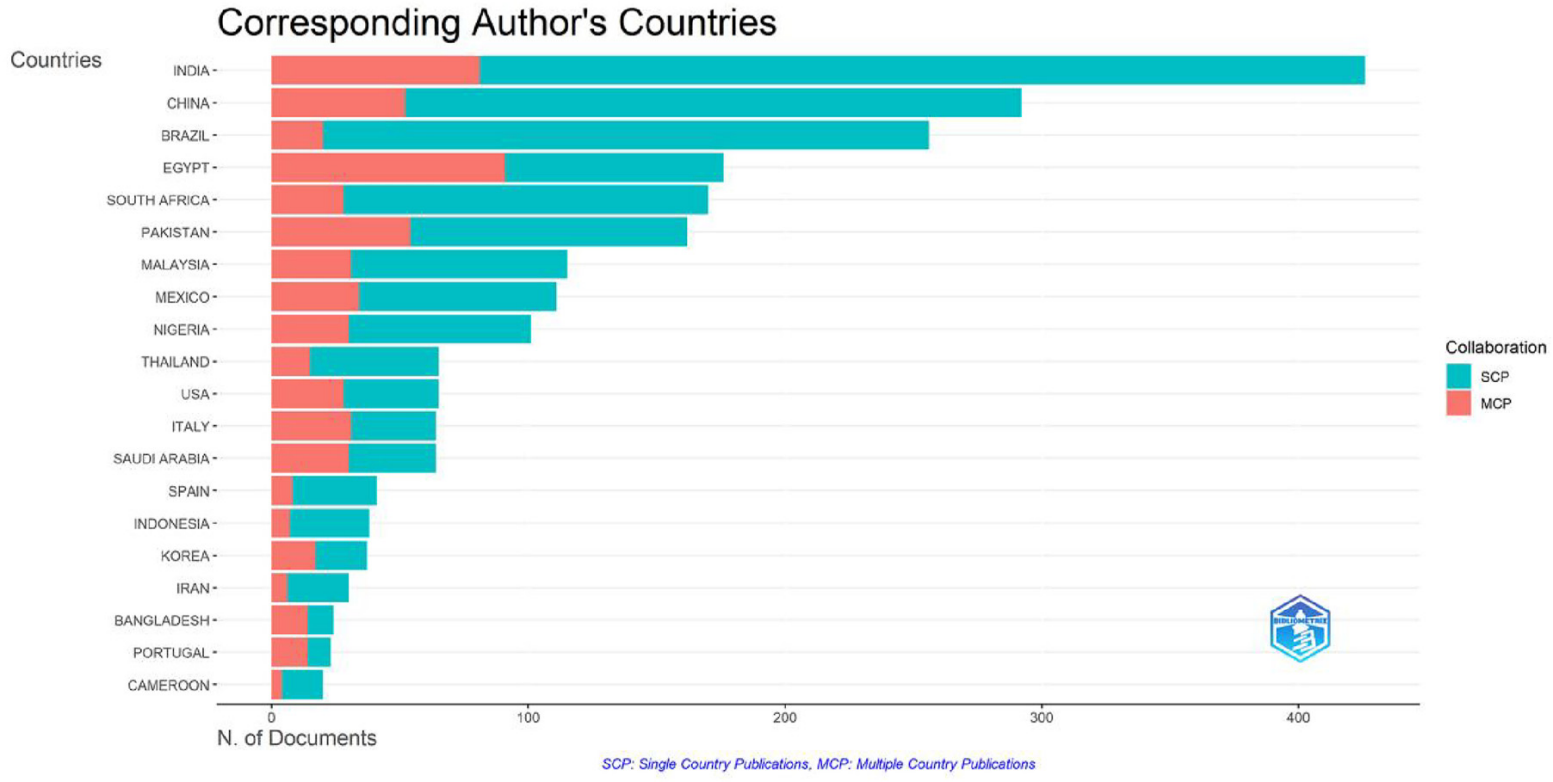
Corresponding author’s countries/regions. This chart displays the top 20 most productive countries/regions and the ratio of their multi-country publications (MCP) to single-country publications (SCP). The length of each bar segment represents the number of documents, with red indicates MCP and blue indicates SCP.

Figure 6 examines the publication patterns of the top 20 most productive countries/regions in *M. oleifera* research, providing insights into global collaboration and knowledge flow. The chart differentiates between single-country publications (SCP) and multi-country publications (MCP), indicating the collaboration strategies of major research entities. International cooperation intensity is measured by the proportion of MCPs, where at least one author’s affiliation differs from the corresponding author’s. The analysis reveals significant differences in international collaboration tendencies. India, with the highest total publications, primarily engages in domestic collaborations, suggesting a relatively mature and potentially self-sufficient research system. Egypt, ranking fourth in total output, exhibits a high MCP ratio, highlighting the significant role of international collaboration in its research activities. China and Pakistan also demonstrate substantial engagement in international collaborations. These findings are crucial for identifying key knowledge nodes and potential collaboration partners. India’s autonomous, high-productivity system positions it as a vital knowledge source and reference. The high network openness of Egypt, China, and Pakistan makes them important partners for accessing the international research network and obtaining diverse resources. Figure 6 provides a basis for scholars to develop tailored international collaboration strategies, such as knowledge exchange with India and collaborative networks with Egypt and others. Including this figure illustrates how an objective analysis of the field’s collaborative landscape can guide scholars in positioning and optimizing their research pathways.

**Figure 7:**
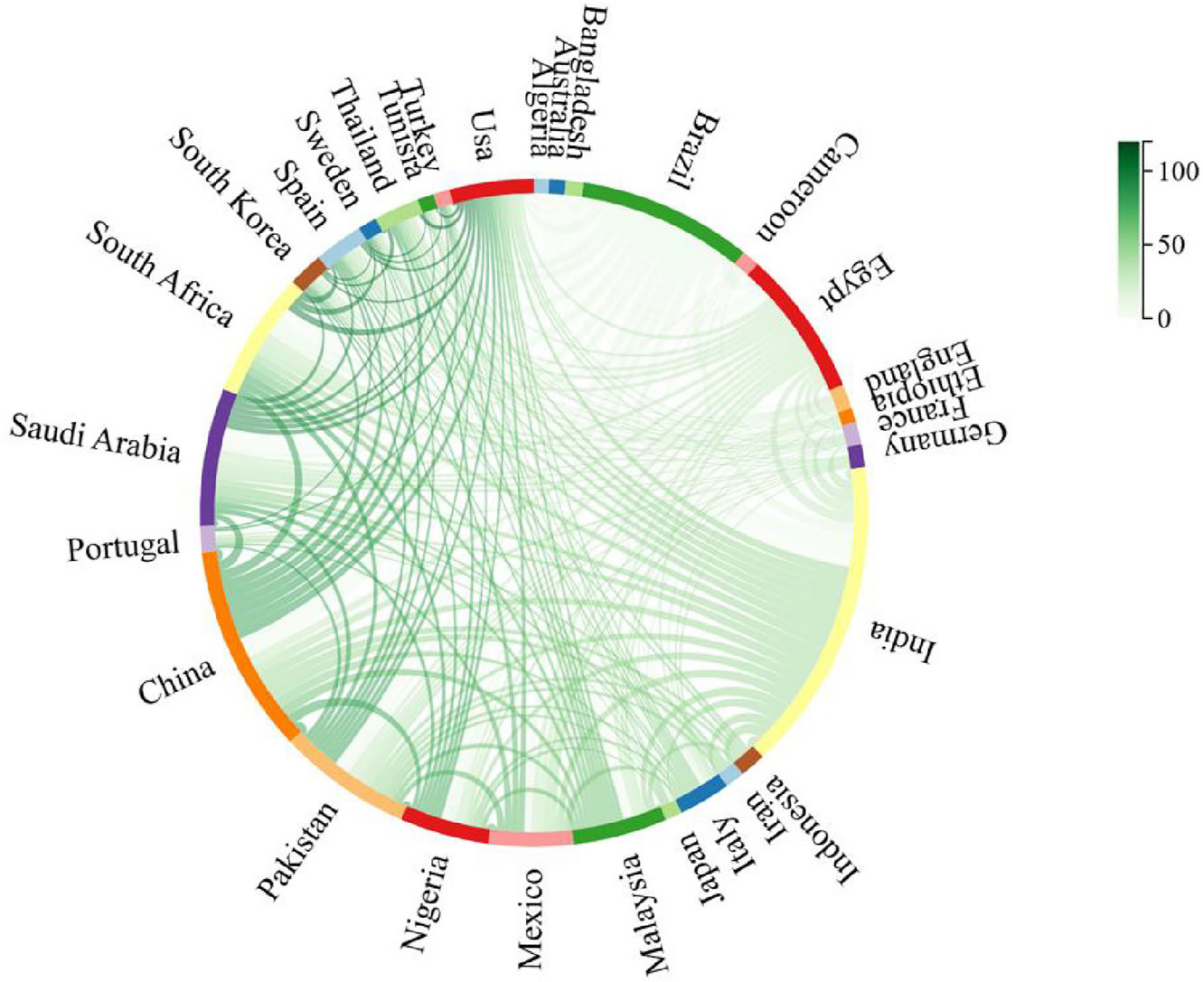
Chord diagram of international cooperation. The length of each colored segment around the circle represents the publication volume of each country/region, with longer segments indicating more publications. The darkness of connecting lines signifies stronger collaboration ties.

Figure 7 utilizes a chord diagram to visualize the publication scale and collaboration network among the top 30 most productive countries/regions in the *M. oleifera* research field. This representation serves not only as a depiction of the collaboration landscape but also as a crucial supplement to our study in understanding the dynamics of global knowledge production and identifying strategic hubs. In the diagram, the arc length corresponding to each country/region on the circle represents its total publication volume, while the thickness and color depth of the lines connecting countries/regions intuitively reflect the strength of their collaborative relationships. The analysis indicates that India and China are not only dominant in terms of publication volume but also confirm their roles as core nodes in the global collaboration network through their extensive and diverse collaborative links, which corroborates the higher degree of international collaboration (MCP) revealed in Figure 6. More specifically, the diagram clearly reveals regional strategic research alliances formed by frequent interactions among countries such as Saudi Arabia, Egypt, Pakistan, and India. Therefore, including Figure 7 in this paper is necessary. It further delineates the specific topological structure of the collaboration network based on the national collaboration tendencies revealed by Figure 6, providing a visual strategic framework for positioning research and identifying potential collaboration pathways within this paper. By integrating information from both figures – that is, a country’s collaboration tendency (Figure 6) and its actual position within the collaboration network (Figure 7) – scholars can more systematically develop differentiated strategies for seeking high-impact international collaborations.

## 6 Main research directions of *M. oleifera*

### 6.1 Citation burst detection of keywords and references

**Figure 8:**
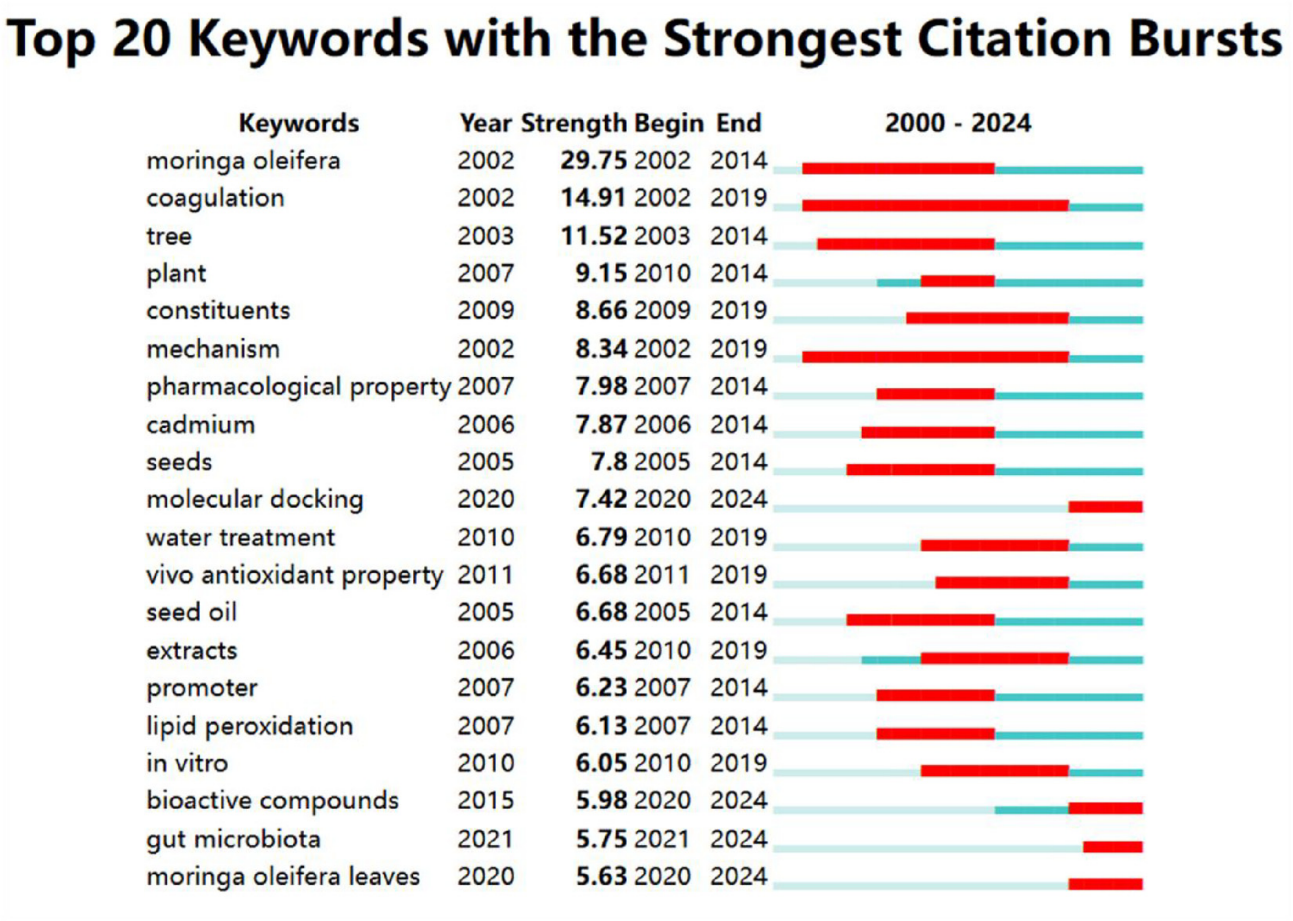
The top 20 keywords in *Moringa oleifera* research with strongest citation bursts. First appearance, burst strength, and burst period for each keyword are indicated. Red bars represent periods of significant attention, with longer lengths indicating longer burst durations.

Citation burst detection analysis reflects the dynamic changes in the frequency of terms, precisely identifying keywords or articles whose citation volume has surged suddenly within a given period. Such bursts of information attract significant attention from the academic community, making them hotspots or cutting-edge topics of research and driving the development of the field. We set the time slicing parameters from January 2000 to December 2024, analyzing each year as a separate slice. We also selected “keywords” and “references” as node types. Additionally, to detect bursts, we applied a g-index with a scaling factor of *k* = 8 for keywords, and set to *k* = 2 for references. Using CiteSpace, we captured and analyzed representative indicators such as burst duration, intensity, and timing. Figure 8 and Table 2 display the top 20 keywords and references with the highest burst strength in *M. oleifera* research, with the strength values highlighted in bold to reveal the extent and changes in their attention.

**Table 2:** The top 20 references in *Moringa oleifera* research with strongest citation bursts.

References	Year	Strength	Begin	End	2000–2024
Leone A, 2015, Int J Mol Sci, V16, P12791-12835, DOI 10.3390/ijms160612791, DOI	2015	34.62	2015	2024	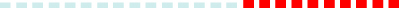
Gopalakrishnan L, 2016, Food Science and Human Wellness, V5, P49-56, DOI 10.1016/J.FSHW.2016.04.001, DOI	2016	31.36	2016	2024	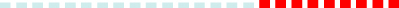
Anwar F, 2007, Phytother Res, V21, P17-25, DOI 10.1002/ptr.2023, DOI	2007	30.57	2007	2014	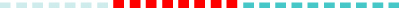
Stohs SJ, 2015, Phytother Res, V29, P796-804, DOI 10.1002/ptr.5325, DOI	2015	26.69	2015	2024	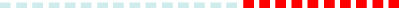
Mbikay M, 2012, Front Pharmacol, V3, DOI 10.3389/fphar.2012.00024, DOI	2012	25.77	2012	2019	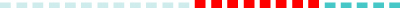
Leone A, 2016, Int J Mol Sci, V17, P2141, DOI 10.3390/ijms17122141, DOI	2016	19.85	2016	2024	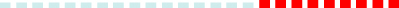
Vergara-Jimenez M, 2017, Antioxidants-Basel, V6, P91, DOI 10.3390/antiox6040091, DOI	2017	19.69	2017	2024	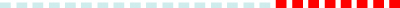
Al-Asmari AK, 2015, Plos One, V10, DOI 10.1371/journal.pone.0135814, DOI	2015	17.78	2015	2024	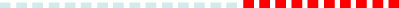
Saini RK, 2016, 3 Biotech, V6, P203, DOI 10.1007/s13205-016-0526-3, DOI	2016	17.58	2016	2024	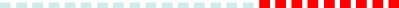
Vongsak B, 2013, Ind Crop Prod, V44, P566-571, DOI 10.1016/j.indcrop.2012.09.021, DOI	2013	17.32	2015	2019	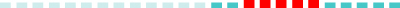
Abd Rani NZ, 2018, Front Pharmacol, V9, DOI 10.3389/fphar.2018.00108, DOI	2018	16.29	2020	2024	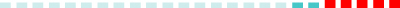
Moyo B, 2011, Afr J Biotechnol, V10, P12925	2011	16.12	2011	2019	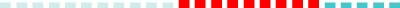
Kou XJ, 2018, Nutrients, V10, P343, DOI 10.3390/nu10030343, DOI	2018	15.89	2020	2024	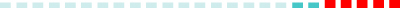
Lakshmipriya Gopalakrishnan, 2016, Food Science And Human Wellness, V5, P49-56, DOI 10.1016/j.fshw.2016.04.001, DOI	2016	15.12	2020	2024	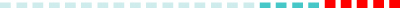
Verma AR, 2009, Food Chem Toxicol, V47, P2196-2201, DOI 10.1016/j.fct.2009.06.005, DOI	2009	14.67	2010	2014	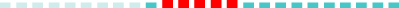
Islam Z, 2021, Int J Food Sci, V2021, DOI 10.1155/2021/6627265, DOI	2021	14.64	2021	2024	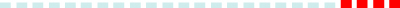
Ghebremichael KA, 2005, Water Res, V39, P2338-2344, DOI 10.1016/j.watres.2005.04.012, DOI	2005	14.32	2005	2014	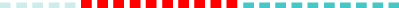
Razis AFA, 2014, Asian Pac J Cancer P, V15, P8571-8576, DOI 10.7314/APJCP.2014.15.20.8571, DOI	2014	14.25	2015	2019	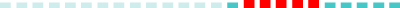
Rodríguez-Pérez C, 2015, Ind Crop Prod, V66, P246-254, DOI 10.1016/j.indcrop.2015.01.002, DOI	2015	13.38	2015	2019	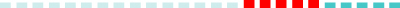
Falowo AB, 2018, Food Res Int, V106, P317-334, DOI 10.1016/j.foodres.2017.12.079, DOI	2018	13.26	2018	2024	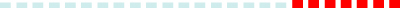

As a core keyword in *M. oleifera* research, “*M. oleifera*” has become a research hotspot since 2002. This is followed by terms such as “coagulation”, “tree”, and “plant”, indicating that research on *M. oleifera* has increasingly focused on the functional development of the plant, particularly its coagulation properties. Meanwhile, Figure 8 shows that the research on “coagulation” and “mechanism” has experienced the longest duration, which indicates that *M. oleifera* holds significant importance in the fields of medicine and chemistry. Additionally, the keywords “molecular docking”, “bioactive compounds”, “gut microbiota”, and “*M. oleifera* leaves” have relatively recent citation bursts and are primarily focused on the pharmacological activities and nutritional components of *M. oleifera*. These areas may emerge as future research hotspots.

The timeline and intensity of each reference’s burst are depicted, with red bars illustrating the period of heightened citation activity.

In Table. 2, we observe that the article published by Leone A. in 2015 [3] has the highest citation intensity and continued to surge to the present day. This article provides a comprehensive review of cultivation, genetics, ethnopharmacology, phytochemistry, and pharmacological research progress of *M. oleifera*, offering a solid scientific basis for its development and utilization. Subsequently, the research by Gopalakrishnan and Anwar [7], 14] has demonstrated nutritional value and medicinal potential of *M. oleifera* as a multifunctional plant. The earliest citation burst was a study published by Ghebremichael KA [51], which centered on the purification and subsequent activity assay of the coagulant protein extracted from *M. oleifera* seeds. The study demonstrated that the seed extract exhibits effective coagulation properties even at low concentrations, making it a promising candidate for large-scale water treatment and the development of *M. oleifera* applications. Additionally, the article published by Stohs SJ in 2015 [52] has the longest burst duration. The study emphasizes that *M. oleifera* is a multifunctional and highly potential plant, and suggests that future research and development of *M. oleifera* extracts should be standardized.

**Figure 9:**
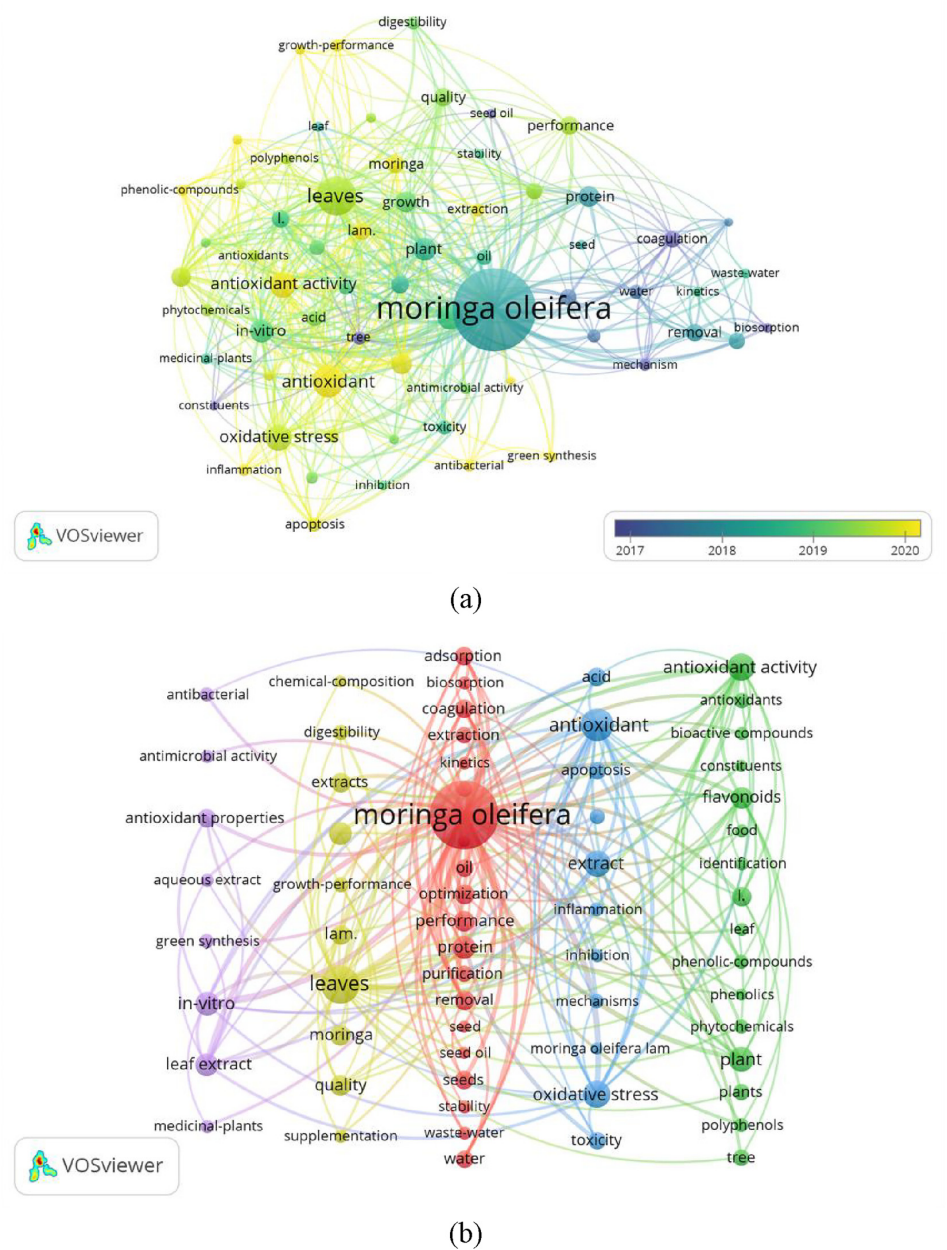
The timeline (a) and clustering (b) visualization of keywords. (a) Timeline view from 2000 to 2024, where node size indicates the number of publications and color denotes the year of publication. (b) Clustered view with five groups: purple for medicinal and clinical functions, yellow for growth-promoting effects, red for bioflocculant research, blue for molecular extraction and mechanisms, and green for nutritional components development and application.

To further reveal the research hotspots and development trends of *M. oleifera*, we visualized and clustered the cited keywords using colors and connecting lines. We established a criterion setting the minimum number of occurrences for a keyword at 30, with 125 out of 10,111 keywords ultimately meeting this threshold. Figure 9(a) shows that over time, new research hotspots in *M. oleifera* studies have continuously emerged and have increased gradually since 2019. Figure 9(b) clusters the keywords into five groups – purple, yellow, red, blue, and green – centered around the core theme of *M. oleifera*, with the latter three clusters being more closely connected. Each cluster represents different research hotspots and application directions of *M. oleifera*.

The purple cluster represents research on the medicinal and clinical functions of *M. oleifera*, with key terms including antioxidant properties, *in vitro*, leaf extract, medicinal plants, and antibacterial. As a medicinal plant, *M. oleifera* is continually being explored for its antioxidant, antibacterial, anti-inflammatory, and anticancer properties. It has demonstrated extensive potential for application in both traditional and modern medicine and is often referred to as the “miracle tree.”

The yellow cluster represents research on the growth-promoting effects of *M. oleifera*, with key terms including leaves, extracts, quality, growth, and digestibility. *M. oleifera* has been shown to promote growth and improve quality in both plants and animals. It can serve as a daily feed and a natural plant growth stimulant for livestock and fish. Moreover, its extracts can enhance the yield and quality of crops, agricultural products, and by-products, thereby improving the livelihoods of farmers and addressing the issue of traditional feed shortages.

The red cluster represents research on *M. oleifera* as a bio flocculant, with key terms including protein, coagulation, adsorption, seeds, and water. *M. oleifera* seeds are rich in cationic proteins and bioactive compounds that exhibit excellent coagulation properties. These properties enable it to effectively remove suspended particles, heavy metal ions, algae, and organic pollutants from water. As a fast-growing plant with high seed yields and easy accessibility, *M. oleifera* is an efficient, environmentally friendly, and cost-effective bioflocculant that is widely used in water treatment.

The blue cluster represents research on the molecular extraction and mechanisms of *M. oleifera*, with key terms including antioxidant, extract, oxidative stress, mechanisms, and apoptosis. Through molecular extraction and cytotoxicity studies, the mechanisms of action of *M. oleifera* in humans and other organisms have been elucidated, thereby ensuring the safety and efficacy of its medicinal and edible applications.

The green cluster represents research on the development and application of the nutritional components of *M. oleifera*, with key terms including antioxidant activity, flavonoids, plant, leaf, and polyphenols. *M. oleifera* is rich in various bioactive compounds, such as flavonoids, phenols, alkaloids, and tannins, especially in its leaves. These chemical components endow *M. oleifera* with high nutrition and medicinal value, making the development and application of its leaf extracts (MLE) an important area of research.

### 6.2 Subject categories analysis

**Figure 10:**
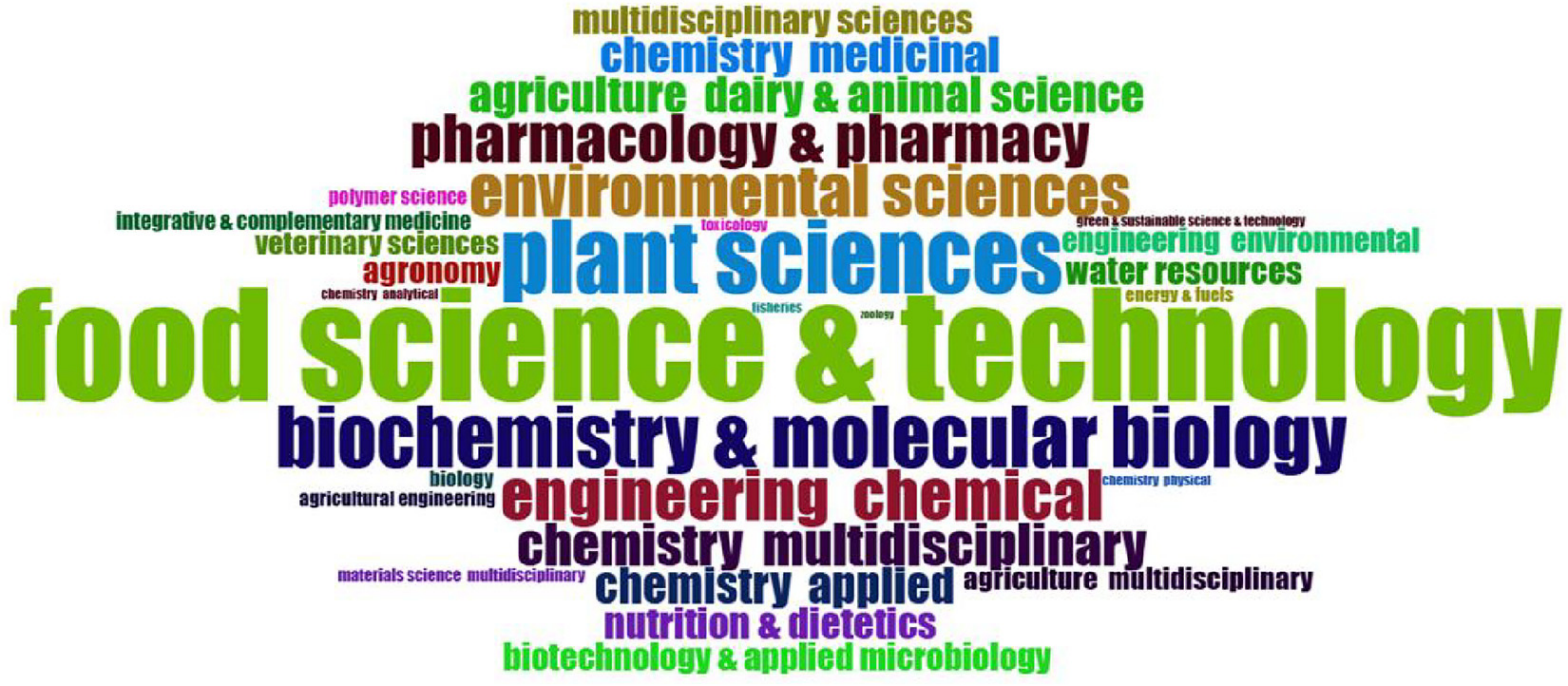
The word cloud includes the top 30 subjects related to *Moringa oleifera*. The size of each term indicates its frequency in the literature, with larger terms representing more frequently studied subject areas.

To further classify the research into specific subject fields, we created Figure 10, which highlights the top 30 subject fields ranked by the number of published papers, with the size of the words and the color intensity of the blocks reflecting the significance of each field in *M. oleifera* research. As can be seen from the figure, the research fields of *M. oleifera* are extensive. Among them, Food Science and Technology is the most prominent area, indicating that food nutrition occupies a dominant position in *M. oleifera* research. Data from Plant Sciences indicate that research on growth, development, genetics, and breeding of *M. oleifera*, as a plant, is also highly significant. Meanwhile, in the field of Biochemistry and Molecular Biology, the bioactive compounds, metabolic pathways, and molecular mechanisms of *M. oleifera* are hotspots of research, which helps to understand its pharmacological effects and nutritional value. Additionally, research on *M. oleifera* extends to other disciplines such as agronomy, energy and fuels, marine biology, and nanotechnology. This demonstrates the interdisciplinary nature of *M. oleifera* research and reflects the widespread applications and research potential of *M. oleifera* as a multifunctional plant across different fields.

### 6.3 Thematic evolution in publications

**Figure 11:**
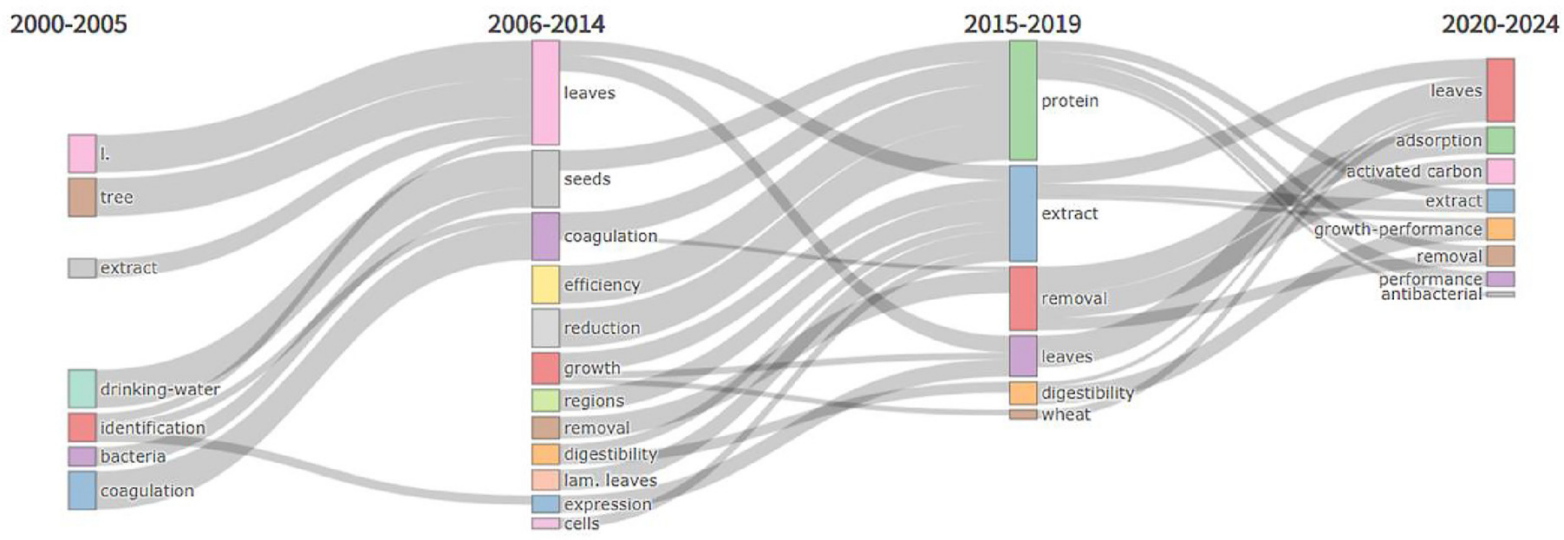
Thematic evolution of *Moringa oleifera*-related publications (2000–2024). Each node represents a research theme, and the length of each node corresponds to the frequency of publications.

In Figure 11, we have utilized the thematic evolution analysis tool from the Biblio-metrix R package to illustrate the progression and interconnections of research topics related to *M. oleifera* across four distinct time periods. The topics are reorganized and refined at different stages, with the research themes on *M. oleifera* continually enriching. Among them, “leaves” has been a constantly focused theme, remaining an important core area from 2006 to 2024. From the first stage to the second stage, the research topics expanded from basic “identification” and “tree” to “coagulation”, “efficiency”, and “growth”, with an increasing focus on the “leaves” and “seeds” of *M. oleifera*. This indicates that both the depth and breadth of the research are increasing. From 2015 to 2019, the research on *M. oleifera* became more concentrated on “protein” and “extract”, as well as the “removal” of pollutants and “digestibility.” This shows an in-depth exploration of the nutritional components and environmental applications of *M. oleifera*, indicating a shift towards more complex application fields. “Protein”, which gathered themes such as “seeds” and “coagulation,” further separated out terms like “antibacterial” in the next stage. This suggests that after focusing on the study of *M. oleifera* proteins, scholars continued to delve into their mechanisms and performance. From 2020 to 2024, new research topics emerged, such as the application of “activated carbon”, “growth-performance”, and “antibacterial.” This may reflect the latest trends and hotspots in *M. oleifera* research.

**Figure 12:**
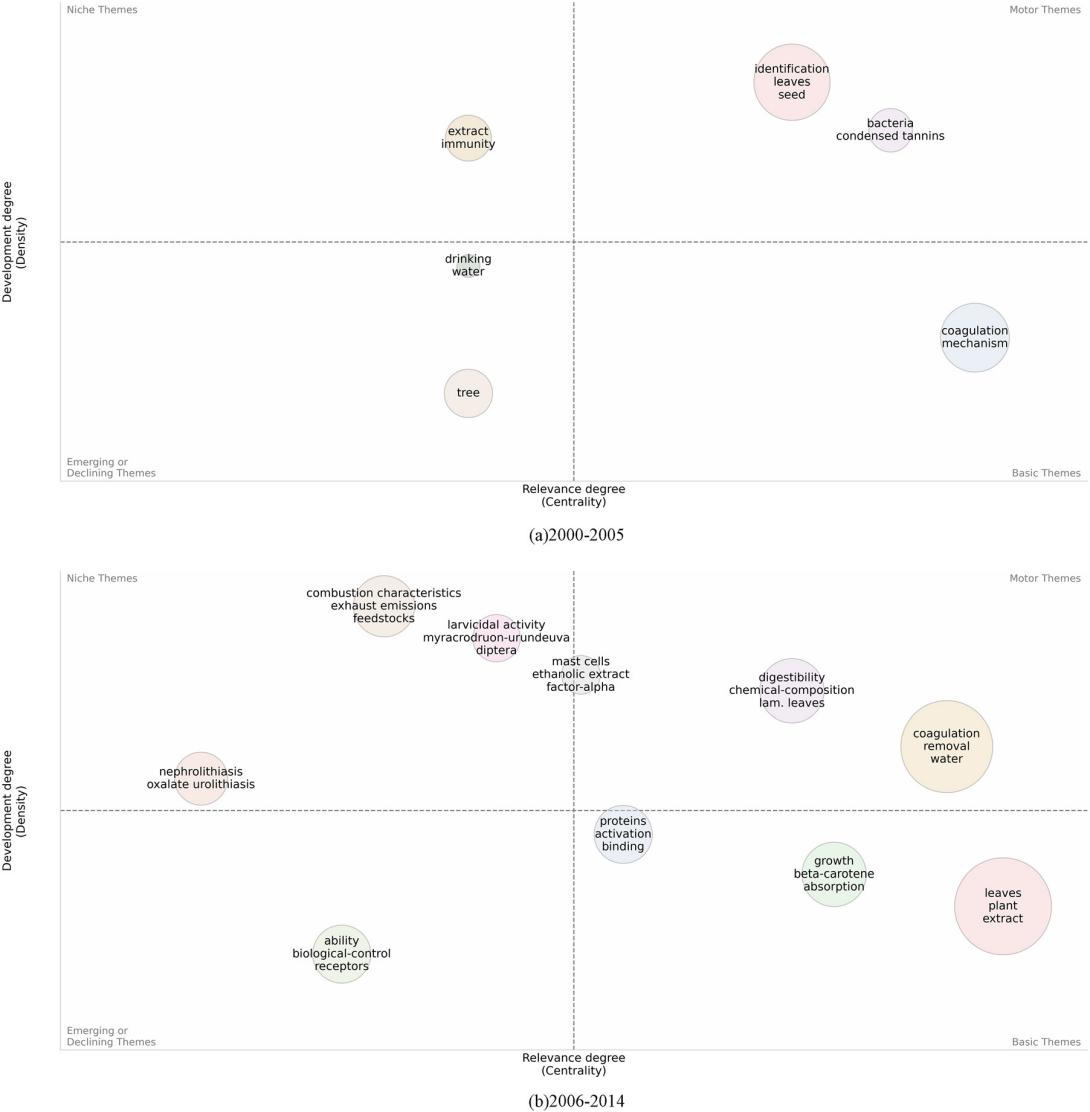

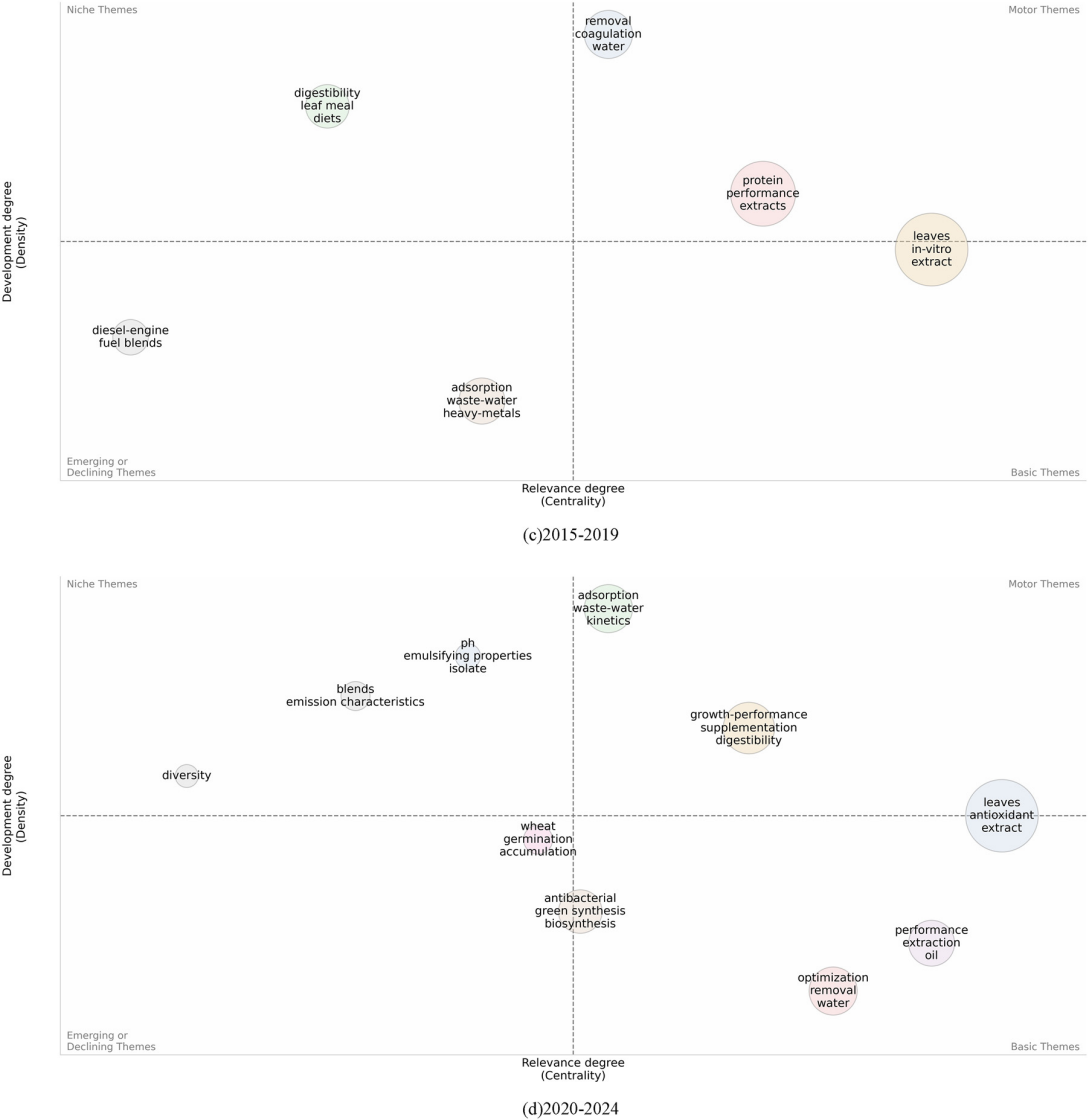
Strategic map of *Moringa oleifera*-related publications (2000–2024). Themes are categorized into four quadrants based on development and relevance. “Motor Themes” (upper right) are active, central areas. “Basic Themes” (lower right) are fundamental and stable. “Niche Themes” (upper left) are specific or emerging. “Emerging or Declining Themes” (lower left) may indicate lesser focus or decline.

To further clarify the specific situations of key themes within each stage, as shown in Figure 12, we have analyzed the four sub-periods separately: (a) 2000–2005, (b) 2006–2014, (c) 2015–2019, and (d) 2020–2024. The size of the circles represents the concentration of the themes, while their positions reflect their relevance. The themes are identified by the most common keywords. In the figure, the themes are divided into four quadrants. The Motor Themes are located in the upper right corner, with high centrality and high density, representing the most active and concentrated areas in *M. oleifera* research and indicating the main direction of the research. The Basic Themes in the lower right corner represent more fundamental and stable areas. The Niche Themes in the upper left corner represent specific or emerging research directions and potential growth points. The Emerging or Declining Themes in the lower left corner may be areas that are less focused on or declining. By analyzing the information within the figures and comparing the differences between them, we can grasp the dynamic changes of different themes.

The research themes on *M. oleifera* have gradually shifted from initial scattered exploration to focusing on specific application areas, and over time, they have continued to expand into new research fields. Since 2000, the identification and development of different plant parts of *M. oleifera* have been a key focus, especially the leaves and seeds. The continuous discovery and extraction of various nutrients and chemical components have made functional development a core research hotspot. Building on the research of leaf extracts, the edible value and pharmacological effects of *M. oleifera* have been well-developed. Meanwhile, the coagulation mechanism of *M. oleifera* in water purification has been continuously optimized, transitioning from a core area to a more mature and stable field. Its role as a biostimulant has also gained increasing attention. Keywords such as “growth-performance supplementation” and “digestibility” are located in the Motor Themes quadrant, confirming that “growth-promotion and quality improvement” constitute the current research core. Conversely, keywords like “biofuels” and “emission characteristics” appear in the Niche Themes quadrant, suggesting that “biofuels and emissions” are emerging as new frontiers. Motor and Basic themes will continue to be research hotspots, while Niche and Emerging or Declining themes may require new research perspectives or technological breakthroughs to regain attention. Future research is likely to place greater emphasis on the application of *M. oleifera* in sustainable agriculture, health foods, and environmental remediation, with a broad outlook for development.

## 7 Discussion

In the preceding analysis, we have systematically traced the evolution of *M*. *oleifera* research from 2000 to 2024. Our study has not only identified the core themes that have been persistently emphasized but also projected feasible future research directions based on our findings. Importantly, our analysis aims to situate research trends within the broader context of scientific and geopolitical developments. This approach is intended to provide a more comprehensive and nuanced understanding of the subject matter, potentially offering additional insights compared to prior biblio-metric analyses.

The citation burst of *M. oleifera* research began in 2002. The seminal work [53] that first reported the significant antioxidant capacity of freeze-dried *M. oleifera* leaf extracts drew widespread attention to the functional properties of *M. oleifera* leaves, making 2003 a peak year for citation rate. The exploration of leaf functions and coagulation applications established 2005 and 2006 significant milestones. Since 2005, continuous verification and optimization by scholars such as Paiva P.M.G., Ghebremichael KA, and Bergamasco R. have enabled the sustainable application of *M. oleifera* in water treatment [51], 54], 55]. Among these, Bergamasco R., one of the most prolific and influential authors, has focused on the active components of *M. oleifera* seeds and their applications in water treatment, particularly in the development of natural coagulants. Her institution, Universidade Estadual de Maringá in Brazil, is also a major contributor institution in the field of *M. oleifera* research. The persistent focus on seed-based coagulation, largely driven by research in water-scarce regions, underscores how local environmental challenges shape global research priorities – a trend well-documented in reviews of plant-based coagulants [56].

The continuous exploration and extraction of nutritional and medicinal properties have positioned it as an important player in the field of Food Science and Technology. This has also fostered closer research and collaboration among developing countries such as India, China, Brazil, Saudi Arabia, and Egypt. A comparative analysis of national research profiles reveals distinct specializations driven by local resources and policies. As outlined in Table 3, our study confirms India’s leading position in publication volume and further identifies its primary focus on the validation of bioactive compounds and their integration into traditional medicine, a trend supported by national policies promoting medicinal plants [57]. China’s formal research on the introduction and cultivation of *M. oleifera* began in 2002 in Yunnan Province [58], establishing China as a main country in *M. oleifera* research and collaboration and making Yunnan Agricultural University the second most productive research institute in this field. The approval of *M. oleifera* as a new food resource in China in 2012, a policy decision, significantly accelerated applied research. Brazilian scholar Anwar F. conducted an in-depth investigation into the multiple medicinal values of *M. oleifera* as a food plant [7], making him one of the authors with a citation burst. King Saud University in Saudi Arabia has achieved a series of important results in combining the bioactive properties of *M. oleifera* with nanotechnology, becoming a high-publication, collaborative institute. This aligns with the kingdom’s strategic vision for local pharmaceutical industries, reflected in current investments in nanotechnology for economic diversification [59]. Egypt’s contributions span a wide range of areas, from basic pharmacological effects to optimization of extraction processes, and its high rate of international co-authorship can be partly attributed to infrastructure initiatives like the Egyptian Knowledge Bank (EKB), which aims to foster scientific collaboration. *M. oleifera* is not only an excellent food crop for addressing malnutrition but is also used as daily feed for livestock in some parts of Africa [60]. Therefore, it attracts continuous attention from the most active journal in the field, the South African Journal of Botany (ISSN: 0254–6299). The divergent research landscape, shaped by national policies, resource endowments, and local challenges, provides a clear strategic framework for guiding future global collaboration in *M*. *oleifera* research. The core of this framework lies in establishing complementary international partnerships based on comparative advantages: for instance, engaging in deep knowledge exchange with India on basic research and traditional medicinal applications; leveraging the “hub” role of countries like Egypt, which show high international co-authorship ratios, to gain access to broader global research networks; and pursuing synergistic exploration with nations such as Saudi Arabia and China on cutting-edge technological integration, including nanotechnology and precision cultivation. This capability-based matching strategy aims to shift international collaboration from sporadic, loosely-connected models towards a more strategic and synergistic global research community.

**Table 3:** Comparative analysis of bibliometric studies on *Moringa oleifera* research.

Feature	Gupta et al. -global [28]	George et al. [64]	Gupta et al. -India [2]	Present study (2025)
Database	Scopus	Scopus	Scopus	Web of Science Core Collection
Time span	1935–2019	2000–2020	1980–2019	2000–2024
Research scope	Global output and long-term trends (85 years)	Collaboration and thematic evolution (21 years)	National case study: India (40 years)	Global output and collaboration, thematic evolution and emerging trends, future directions (25 years)
Key analytical focus	– Publication/citation metrics; – Leading countries/institutions; – Subject category distribution; – High-impact papers	– Co-authorship networks; – Keyword co-occurrence; – Thematic evolution; – International collaboration patterns	– India’s publication landscape; – Leading domestic institutions/authors; – Research quality (citations); – International co-authorship	– Output and collaboration networks of authors, institutions, journals, and countries/regions; – Keyword cluster analysis; – Thematic evolution; – Emerging sustainable application areas
Primary findings	– 3,187 publications; 16.18 % AAGR; – India, Nigeria, Brazil lead; – Dominant fields: Agricultural and Biological Sciences, Pharmacology; – Key journal: S. Afr. J. Bot	– 2,345 publications; – India leads, Brazil, Nigeria follow; – Identified 15 thematic clusters (e.g., Pharmacology, Water Treatment, Biofuel); – U.S. has most international links	– 819 Indian publications; – CFTRI-Mysore, University of Calcutta are top institutions; – Leading field: Pharmacology, Toxicology and Pharmaceutics; – International co-authorship rate: 10.38 %	– 2,704 publications, interdisciplinary; – India’s leadership confirmed; China is a major research country; Egypt has the highest international co-authorship ratio; – Hot keywords: leaves, extraction; – Nutritional components, medicinal uses, and water purification are mature and stable areas; growth promotion and quality improvement are core themes; – Biofuel and emissions are emerging research areas
Identified hotspots	Antioxidants, water treatment	Pharmacology, biochemistry	Medicinal value, phytochemistry	Leaves, extraction, nutritional components, growth promotion, biofuel and emissions
Novel contribution	Established baseline metrics and historical trajectory	Pioneered VOS-viewer analysis of collaborative networks and thematic dynamics	Provided in-depth quantitative national assessment of India’s research ecosystem	– Extended timeline; used VOS-viewer, Cite-Space, and Biblio-metrix R-package to capture the latest trends and diversification phase; – Identified biofuel and growth promotion as emerging sustainable applications; – Proposed future research on *M. oleifera* applications in sustainable agriculture, health foods, and environmental remediation
Limitations and future directions (cited from documents)	Limitation: “the study did not deeply analyze the collaborative patterns among countries” → Our response: integrated a chord diagram and MCP/SCP analysis to decode network topology, hub roles, and national collaboration strategies	Future direction: highlighted the need to analyze collaboration networks to identify strategic hubs → Our response: directly addressed this by mapping hubs (e.g., India, China) and gateways (e.g., Egypt), providing a strategic framework for partnership building	Limitation: constituted a “detailed national case study (India)” without a comparative multinational perspective → Our response: embedded India’s case within a comparative analysis of multiple leading nations (India, China, Egypt, et al.), highlighting unique specializations and roles	–

Our analysis, covering literature up to 2025, provides a more current perspective than previous reviews (see Table 3) and allows for a focused examination of the latest five-year trends. Future research is likely to place greater emphasis on the applications of *M. oleifera* in sustainable agriculture, health foods, and environmental remediation. This trend is corroborated by recent studies. For instance, Barkat et al. highlighted *M. oleifera* leaves, with their exceptional nutritional, medicinal, and commercial value, offer substantial support to sustainable agricultural initiatives. [61]. Meanwhile, Kehong Liang et al. emphasized its significant value in health foods due to the exceptional nutritional richness of its leaves and seeds [62]. Furthermore, Priya Kumari et al. indicated that *M. oleifera* significantly contributes to environmental remediation by absorbing pollutants and CO_2_, purifying water through its seeds, and reducing soil toxins, thereby enhancing ecosystem health and sustainability [63]. The consensus reached in these recent publications strongly aligns with our conclusion, pointing towards the growing research and application interest in *M. oleifera*.

Our extended timeline indicates that *M. oleifera* research is experiencing a phase of diversification and expansion into new interdisciplinary applications. Currently, *M. oleifera* research is still in a period of rapid development, with leaves and extraction remaining hot keywords. The sustained focus on leaves is attributed to their accessibility and richness in bioactive compounds, making them the central subject linking nutritional, medicinal, and functional properties. Meanwhile, extraction is the fundamental step to unlock these bioactive compounds for practical applications. Thus, the frequent co-occurrence of these keywords highlights the core research progression from raw material characterization to functional compound acquisition. Research on nutritional components, medicinal uses, and coagulation for water purification has become more mature and stable. Meanwhile, the application of *M. oleifera* in promoting biological growth and improving quality is gradually shifting from a niche area to a core field and will be a hot topic in the coming period. As the functions of *M. oleifera* continue to expand, the demand for it is also increasing. Therefore, cultivation and production research, as the basis for sustainable supply of *M. oleifera*, has broad application prospects but is still insufficient at present. The emergence of keywords such as “blends emission characteristics”, “digestibility”, and “activated carbon application” indicates that *M. oleifera* research is expanding into new areas, such as environmental remediation. These emerging niches, particularly in biofuel and emission reduction, represent a significant novel insight from our analysis, not highlighted in earlier bibliometric studies [2], 64].

Despite the rarity of bibliometric reviews on *M. oleifera*-related research, we can still compare the data and results obtained from different databases, time periods, and analytical software. This not only allows for a more comprehensive evaluation of *M. oleifera* research also demonstrates the diversity of biblio-metric applications. Through comparison, as illustrated in Table 3, we found that our analysis corroborates key findings from Gupta [2], 28] and George [64] – such as India’s leadership and core research areas – while uniquely identifying the emergence of sustainable application niches like biofuel and growth promotion, thereby confirming the reliability and innovativeness of our results. For example, all studies identified lists of highly productive researchers, confirming India’s leading position in *M. oleifera* research, although the specific rankings may differ. We also found that the research areas of *M. oleifera* are mainly concentrated in pharmacology, food and nutritional uses, as well as its applications in water treatment, environmental management, and biofuel production. Over time, the top-ranked active participants and their collaborations continue to make efforts, while new themes and fields have also emerged to attract our attention.

**Limitations**. While our analysis has provided valuable insights, it also reveals the inherent limitations of bibliometric methods, which must be critically discussed. First, our study is confined to the Web of Science (WoS) database. While WoS is highly regarded, it may exclude relevant research from other databases (e.g., Scopus, regional databases), potentially narrowing the scope of our findings and introducing a source selection bias. Second, our analysis is limited to English-language publications, which may overlook valuable contributions from non-English-speaking regions, leading to a potential geographic bias. Additionally, our search strategy relied solely on titles, which could have excluded relevant studies with ambiguous or non-representative titles. This choice of focusing on articles and reviews, while common, may have inadvertently omitted other types of literature, such as conference papers and book chapters, which could also contain valuable insights. Finally, bibliometric methods primarily measure publication quantity and citation impact rather than intrinsic research quality or broader societal significance. Citation counts and publication venues do not always fully reflect a study’s true impact.

**Future research**. Based on these limitations, we recommend that future research adopt the following measures: To ensure the comprehensive identification of relevant literature, a multilingual and multi-database search strategy should be employed. Moreover, focusing on the growth promotion and quality enhancement of *M*. *oleifera* can increase its application value in agriculture and the food industry. Furthermore, additional research is needed to evaluate the potential applications of *M*. *oleifera* in the fields of biofuel and emissions. Lastly, future research should use more comprehensive methods to assess the quality and impact of research, such as altmetrics or content analysis to gauge societal attention, alongside expert review to evaluate scientific rigor and application potential.

## 8 Conclusions

The present study conducted a bibliometric analysis of 2,704 publications on *M. oleifera* research from 2000 to 2024, revealing a steady growth trajectory with significant acceleration over the past five years. First, research on *M. oleifera* remains in a phase of rapid development, primarily driven by interdisciplinary collaborations among developing countries such as India (426 publications) and China (292 publications). Among key contributors, Bergamasco R. stands out with the highest h-index and g-index, reflecting her influential work on seed-based coagulation for water treatment. Institutionally, Egyptian Knowledge Bank (EKB) has positioned Egypt as the country with the highest proportion of internationally co-authored publications, establishing it as a strategic hub within global research networks, while the South African Journal of Botany serves as a key platform for scholarly exchange.

Citation burst detection and thematic evolution analysis, conducted using VOS-viewer, Cite-Space, and Biblio-metrix R-package, identified “leaves” and “extraction” as persistent research hotspots, underscoring the core research progression from raw material characterization to functional compound acquisition. Notably, studies on nutritional components, medicinal uses, and water purification have matured, whereas growth promotion and biofuel applications have emerged as novel frontiers – a key insight not emphasized in previous bibliometric studies. Keywords such as “blends emission characteristics” and “activated carbon application” signal an expansion into environmental remediation and sustainable energy.

In summary, this analysis projects a growing role for *M. oleifera* in sustainable agriculture, health foods, and environmental remediation. It further proposes that future collaboration strategies should be capability-based, such as leveraging Egypt’s international networks for broader resource access. The findings affirm the enduring value of *M. oleifera* as a focal point for interdisciplinary research, with emerging fields like biofuel applications and environmental sustainability offering new impetus for global synergy.
